# Sodium Valproate-Induced Encephalopathy: A Case Report

**DOI:** 10.7759/cureus.115362

**Published:** 2026-08-28

**Authors:** Sooraj Viswanathan, Syeda Ambreen Fatima, Parisa Moshfeqa, Norhan Shaykhon, Nimrata Lohana, Chander Bhan

**Affiliations:** 1 Internal Medicine, Withybush General Hospital, Haverfordwest, GBR; 2 Internal Medicine, Queen's Hospital, London, GBR; 3 Internal Medicine, Glangwili General Hospital, Carmarthen, GBR; 4 Medicine and Surgery, Royal College of Surgeons in Ireland, Manama, BHR; 5 Cardiology, Withybush General Hospital, Haverfordwest, GBR

**Keywords:** atrioventricular block, drug toxicity, encephalopathy, non-hyperammonemia, sinus bradycardia, valproate, valproic acid

## Abstract

Valproic acid is widely used in neurological and psychiatric practice and is generally well tolerated; however, valproate-induced encephalopathy (VIE) is a rare but clinically significant adverse reaction that may occur even at therapeutic serum concentrations and in the absence of hyperammonaemia or hepatic dysfunction. We report the case of a 63-year-old woman on long-term valproic acid therapy who presented with progressive gait instability, fluctuating consciousness, and marked sinus bradycardia. Neuroimaging, cerebrospinal fluid analysis, and metabolic investigations were unremarkable, with normal serum ammonia and valproate levels within the therapeutic range. Electroencephalography demonstrated generalised cerebral dysfunction without epileptiform activity. Discontinuation of valproic acid led to rapid and complete resolution of both the encephalopathy and the bradycardia, supporting a direct causal relationship. VIE may occur via hyperammonaemic or normoammonaemic mechanisms; this case exemplifies the latter, demonstrating that normal ammonia levels must not be used to exclude the diagnosis. The concurrent sinus bradycardia is an atypical feature rarely described outside the context of overdose, and its co-resolution with the encephalopathy following drug withdrawal further supports a valproate-mediated aetiology. This case highlights that serious neuro-cardiac adverse effects traditionally associated with valproate toxicity may arise during routine therapeutic use, and that prompt recognition and withdrawal of valproate can be curative.

## Introduction

Valproic acid (VPA), first introduced as an antiepileptic agent in the 1960s, is now recognised as a broad-spectrum neuropsychiatric medication used in the management of epilepsy, bipolar affective disorder, and migraine prophylaxis [1,2]. It is among the most widely prescribed antiepileptic drugs globally and is generally well tolerated across a broad age range. Known adverse effects span a wide severity spectrum, from minor gastrointestinal disturbance and tremor to serious complications including hepatotoxicity, haemorrhagic pancreatitis, thrombocytopenia, teratogenicity, and encephalopathy [2,3].

Valproate-induced encephalopathy (VIE) is a rare but potentially life-threatening complication that may arise at any stage of treatment, including during long-term maintenance therapy [1,4]. VIE most frequently occurs in association with elevated serum ammonia levels, a phenomenon thought to be related to VPA-induced disruption of mitochondrial beta-oxidation, depletion of L-carnitine stores, and subsequent impairment of hepatic urea cycle function [4-6]. However, non-hyperammonaemic presentations have also been described and may pose a particular diagnostic challenge, as they can occur despite therapeutic serum valproate concentrations and normal liver function tests [1,7,8].

The pathophysiology underlying non-hyperammonaemic VIE remains incompletely understood. Several mechanisms have been proposed, including increased astrocytic glutamine accumulation leading to osmotic stress, direct mitochondrial dysfunction, carnitine depletion, and individual genetic susceptibility; however, the relative contribution of these factors has not been fully established [1,8]. Identified risk factors for VIE include polypharmacy, particularly with agents that may enhance central nervous system (CNS) depression or affect ammonia metabolism, high initial dosing, prolonged therapy, and metabolic stressors such as fasting [1,2].

Clinical manifestations range from subtle cognitive slowing and behavioural change to profound impairment of consciousness, and onset may be insidious or subacute, particularly in patients receiving therapeutic doses [3,9]. Diagnosis may be delayed when routine investigations, including serum ammonia, serum valproate concentrations, liver function tests, and neuroimaging, are unrevealing [1,3,7].

Cardiac manifestations associated with valproate therapy have been reported most often in the setting of acute toxicity or overdose, where findings such as sinus bradycardia, QT interval prolongation, and hypotension have been described [10-12]. Evidence for clinically significant cardiac effects during therapeutic use is limited and consists largely of isolated case reports and small observational studies, which have suggested possible effects on cardiac repolarisation and atrial electrophysiology [11,13]. Consequently, the relationship between therapeutic valproate exposure and cardiac conduction abnormalities remains incompletely characterised. When neurological deterioration and cardiac abnormalities occur concurrently in patients receiving long-term valproate therapy, a drug-related aetiology may not be immediately recognised, potentially contributing to diagnostic delay.

We describe a case of progressive neurological deterioration accompanied by marked sinus bradycardia in a patient receiving long-term VPA therapy, despite normal serum ammonia and therapeutic valproate levels, with resolution of both neurological and cardiac manifestations following drug withdrawal.

Verbal informed consent was obtained from the patient prior to publication. This work was previously presented as an oral case presentation at the National Conference by the Dhaka Medical College Alumni Association UK on 7 February 2025.

## Case presentation

A 63-year-old female presented to A&E (Accident and Emergency) by ambulance following a motor vehicle accident while driving at 20 km/h. Witnesses reported she had been swerving across the road as if distracted prior to the accident. On paramedic assessment, she was hypoglycaemic with a blood glucose level of 3.6 mmol/L (4-11 mmol/L), markedly bradycardic, and appeared confused, unable to answer basic questions about her own health. Mild hypoglycemia on presentation was unlikely to be the primary driver of neurological deterioration, as glucose correction did not resolve the confusion, and encephalopathy persisted and worsened despite subsequent normal glucose readings.

She described progressive imbalance over the preceding two months, with multiple falls. Three months earlier she had attended the emergency department after an unwitnessed fall; a CT head scan was unremarkable, but she self-discharged before full assessment. She reported two additional falls in the intervening period.

Her medical history included bipolar affective disorder with recurrent manic episodes, anxiety and depression, giant cell arteritis, folate-deficiency anaemia and chronic auditory hallucinations. Regular medication includes mirtazapine, valproic acid, omeprazole, prednisolone and alendronic acid. The patient had been receiving valproic acid therapy for four years prior to admission.

On admission, clinical examination was unremarkable with GCS (Glasgow Coma Scale) of 15/15 and no focal neurological deficits. Cranial nerve testing, motor and sensory examination, and cerebellar signs were normal. There were no signs of meningism. Routine laboratory investigations demonstrated normocytic anaemia (chronic), mild leukopenia (3.3 × 10⁹/L) with neutropenia (1.4 × 10⁹/L), an elevated C-reactive protein (74 mg/L), and a mildly elevated complement C3 (1.74 g/L). The remaining blood investigations, including autoimmune serology, were unremarkable. Cerebrospinal fluid (CSF) analysis, including biochemistry and an infective polymerase chain reaction (PCR) panel, was normal (Table 1).

**Table 1:** Blood and CSF results Noted valproic acid and ammonia levels within the normal and acceptable range. Hb: haemoglobin; MCV: mean corpuscular volume; CRP: C-reactive protein; tTG: tissue transglutaminase; TPO: thyroid peroxidase; CSF: cerebrospinal fluid; PCR: polymerase chain reaction

Test	Result	Reference Range
Hb	93 g/L	115-165 g/L
MCV	98 fL	80-100 fL
WBC	3.3x10^9/L	4.0-11.0x10^9/L
Neutrophils	1.4x10^9/L	1.7-7.5x10^9/L
CRP	74 mg/L	<5 mg/L
Bilirubin	9 umol/L	<21 umol/L
Protein	67 g/L	60-80 g/L
Albumin	36 g/L	35-50 g/L
Alkaline phosphatase	60 g/L	30-130 g/L
Alanine transaminase	<5 U/L	<33 U/L
Ammonia	<10 umol/L	<50 umol/L
Valproic Acid	104 mg/L	50 - 125 mg/L
Cortisol	433 nmol/L	> 420nmol/L
Creatine Kinase	26 U/L	25-200 U/L
Complement C3	1.74 g/L	0.75-1.65 g/L
Complement C4	0.35 g/L	0.14-0.54 g/L
Anti-IgA tTG Antibodies	<0.1 U/ml	0.0-10.0 U/ml
Anti-TPO Antibodies	8.8 U/ml	<34.0 U/ml
Anti-Acetylcholine Receptor Antibodies	Negative	-
CSF Lactate	1.7 mmol/L	<3.0 mmol/L
CSF Protein	0.40 g/L	0.15-0.45 g/L
CSF Glucose	2.8 mmol/L	2.2-3.9 mmol/L
CSF Herpes Simplex Type 1 PCR	Negative	-
CSF Herpes Simplex Type 2 PCR	Negative	-
CSF Varicella Zoster PCR	Negative	-
CSF Meningococcal PCR	Negative	-

**Figure 1:**
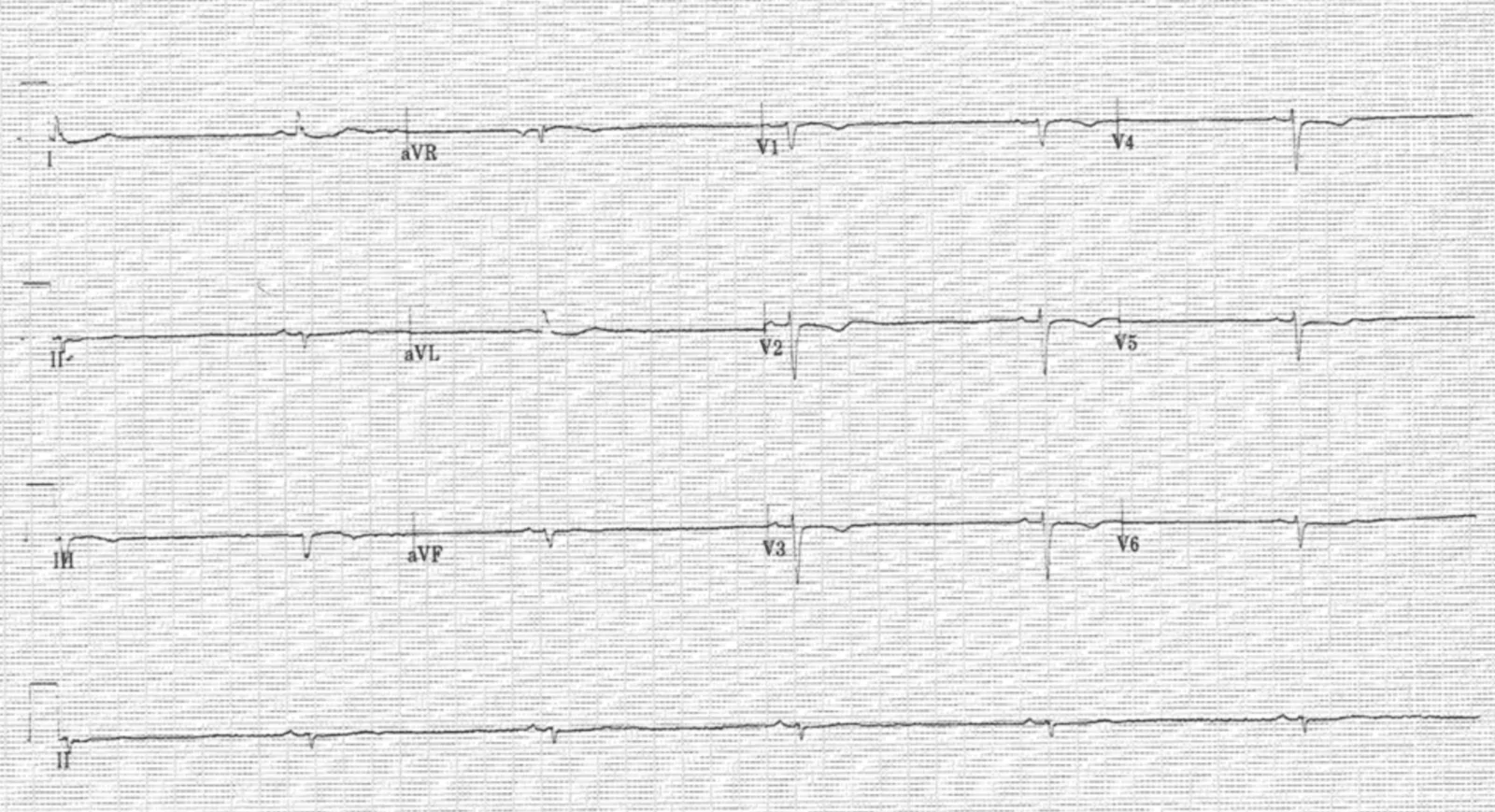
12-Lead ECG Marked sinus bradycardia. Left axis deviation. VT rate: 34 bpm; PR interval: 152 ms; QRS duration: 90 ms; QT/QTc: 462/347 ms

**Figure 2:**
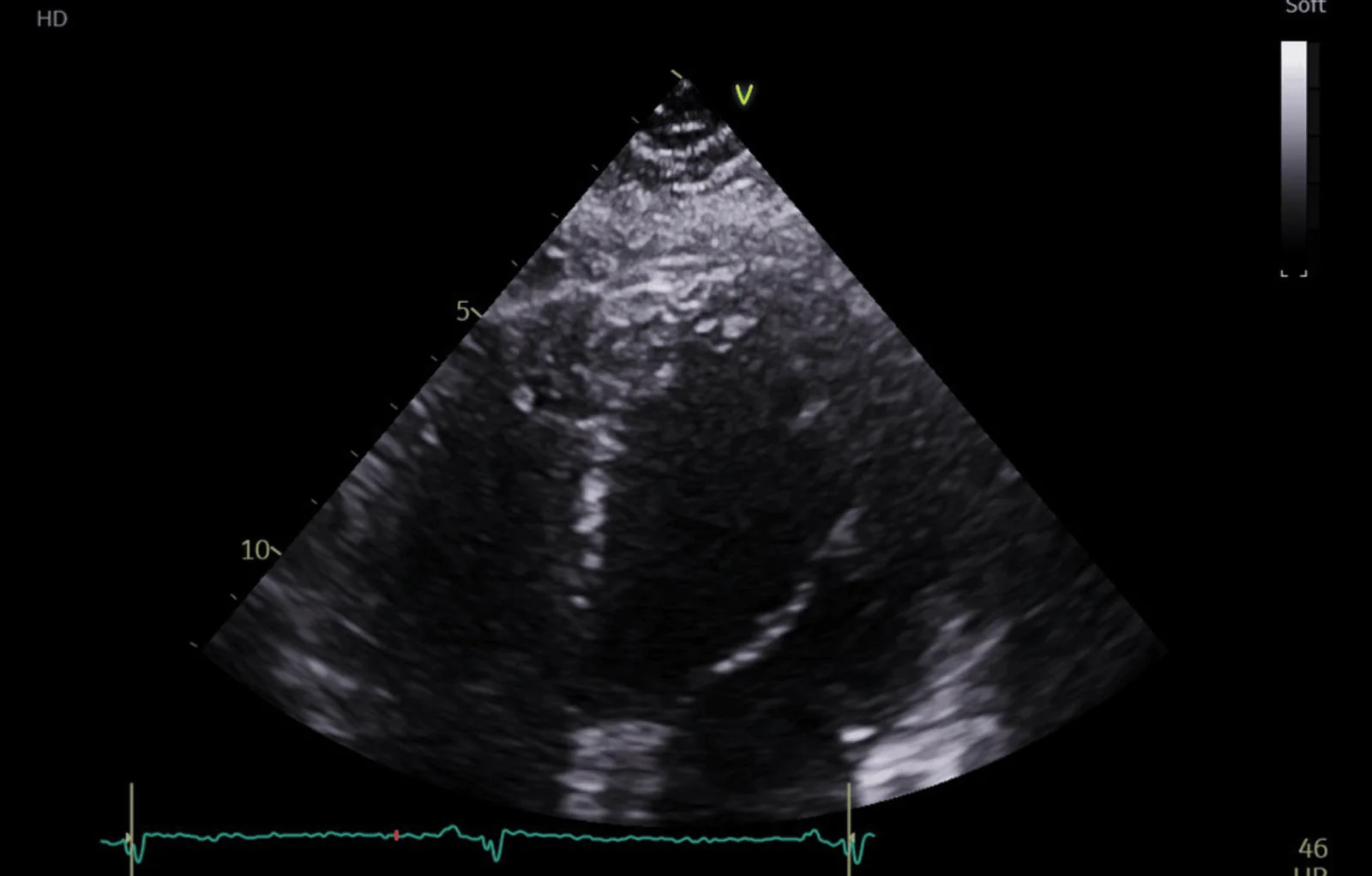
Transthoracic echocardiogram Transthoracic echocardiogram showing no regional wall motion defects or other abnormalities.

The patient was hemodynamically stable, and, therefore, did not require isoprenaline or additional cardiac support during admission. A 12-lead ECG showed sinus bradycardia (HR 34 bpm), which was not present before this admission (Figure 1). Transthoracic echocardiography showed no structural abnormalities or regional wall motion defects (Figure 2). No chest pain was reported during admission. A cardiac MRI was arranged as an outpatient, which did not reveal any abnormality.

On presentation, the patient was fully conscious with a GCS score of 15/15. During admission, her neurological status progressively deteriorated. By day 11 of admission, her GCS had deteriorated to 12/15 (E3V3M6), accompanied by poor oral intake and worsening gait instability. Her condition continued to worsen, and by day 18 her GCS had fallen to 8/15 (E2V1M5). At this stage, she was unable to hold a glass of water or sustain a conversation for more than a few minutes and experienced intermittent brief episodes of loss of consciousness lasting several seconds. Owing to the significant decline in her level of consciousness and inability to maintain adequate oral intake, nasogastric tube feeding was being considered. She also developed urinary incontinence on day 18, necessitating urinary catheterisation.

The patient was commenced on acyclovir intravenously 700 mg three times a day for suspected viral encephalitis on day 18 while awaiting lumbar puncture results.

**Figure 3:**
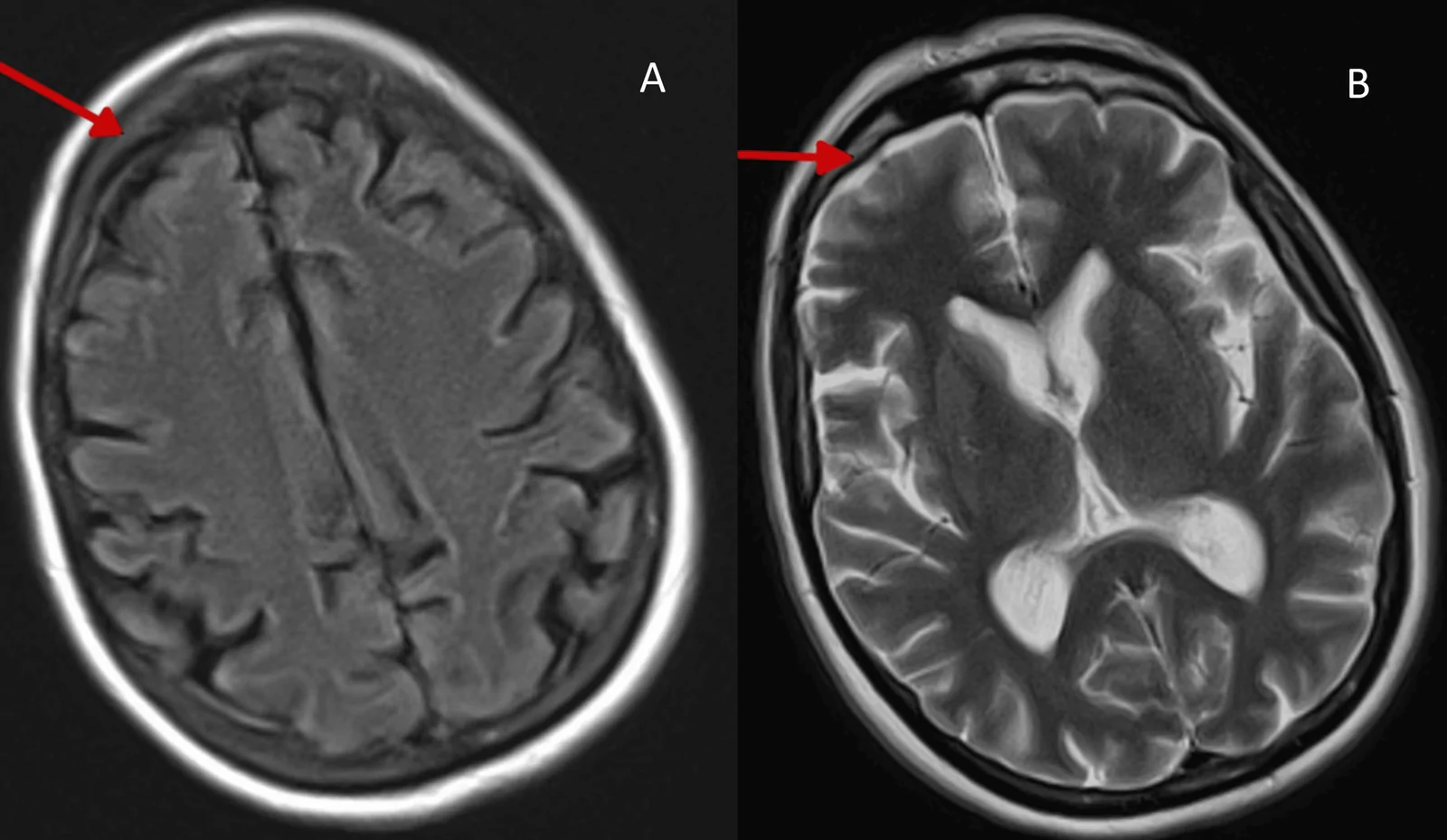
CT Head (A) and MRI Brain (B) A: No acute features. There is significant generalised volume loss for a patient of this age. B: No acute abnormal findings. Generalised parenchymal volume loss is indeterminate for the cause.

Computed Tomography Head (Figure 3A) on day 2 and MRI Brain on day 19 (Figure 3B) imaging demonstrated no acute abnormalities. Outpatient electroencephalography (EEG) following discharge concluded underlying generalised brain dysfunction with focal attenuation in brain regions corresponding to temporal recording areas, left more than right. No epileptiform EEG abnormalities had been recorded.

Given ongoing depressed consciousness, valproic acid and mirtazapine were withheld on day 19 of this admission. Following cessation of medication, the patient's GCS returned to baseline of 15/15 and sinus bradycardia resolved within 24 hours.

Valproic acid was subsequently reintroduced on day 21 of admission at a reduced dose of 250 mg once daily, compared with her pre-admission dose of 1250 mg/day (500 mg in the morning and 750 mg in the evening). Following reintroduction, she remained neurologically stable with a sustained GCS of 15/15 and no further episodes of neurological deterioration. She was discharged on day 27 with arrangements for outpatient psychiatric follow-up the following month.

The care of this patient was co-ordinated through a multidisciplinary team of psychiatrists, neurologists, stroke specialists, endocrinologists, nurses, physiotherapists and dietitians.

## Discussion

Valproic acid is a widely used antiepileptic and mood-stabilising agent, also prescribed for migraine prophylaxis and other psychiatric conditions [1, 2]. Although generally well tolerated, valproate therapy has been associated with a spectrum of adverse effects ranging from mild gastrointestinal symptoms and tremor to serious complications such as hepatotoxicity, pancreatitis, and encephalopathy [2,3]. A rare but important adverse reaction is valproate-induced encephalopathy, which is frequently associated with hyperammonaemia [1,4,5].

This patient has been taking VPA for diagnosed bipolar disorder for the last 4 years, with raised risk factors of VIE being polypharmacy and co-existence of mirtazapine, a CNS depressant which can potentiate neurotoxic effects of VPA. Reported risk factors for developing valproate-induced encephalopathy are usually noted as high initial dose, long-term therapy, polypharmacy, or a state of fasting [1,2]. A recent case series of valproate-induced hyperammonaemia reported neuropsychiatric manifestations including mental confusion, irritability, slurred speech, and coordination and balance disorders, highlighting that early neurological features of valproate toxicity may be subtle and non-specific, potentially contributing to delayed recognition in clinical practice [14].

Pathogenesis of valproate-induced encephalopathy is enigmatic, with existing literature linking increased levels of ammonia with the reduction of consciousness, which is contradictory to this patient's normal ammonia levels. A proposed mechanism for non-hyperammonaemic VIE is increased glutamine production and decreased glutamine release by astrocytes, resulting in glutamine accumulation, increased intracellular osmolarity, astrocyte swelling, and cerebral oedema [1,5,6,7]. Because serum or CSF glutamine was not investigated, it cannot be concluded that glutamine accumulation caused the encephalopathy. An additional hypothesis suggests mitochondrial toxicity, carnitine depletion, and genetic susceptibility can contribute to VPA-induced encephalopathy irrespective of serum levels [1,8]. Valproate can rarely cause non-hyperammonaemic encephalopathy with parkinsonian features, with 28 reported adult cases resolving after drug withdrawal [9]. 

In VPA overdose, onset of symptoms is typically acute, and features of reduced consciousness, seizure and cardiorespiratory depression are often seen [1,10]. In contrast with therapeutic dose symptoms of toxicity are usually subacute in onset and can present as cognitive slowing, unsteadiness, behavioural change or confusion. Despite therapeutic valproate levels, the patient developed encephalopathy, initially presenting with progressive unsteadiness and falls which were likely to be early signs. Other causes were excluded, and symptoms resolved rapidly after stopping valproate, strongly supporting the diagnosis [1]. In short, subacute and less dramatic presentation in therapeutic doses of VPA can cause delayed diagnosis in comparison to cases of obvious overdose. In one case report, the patient had neither hyperammonaemia nor L-carnitine deficiency, and despite subtherapeutic valproate levels, consciousness improved after its discontinuation [7].

Reported cases of valproate-induced encephalopathy reported high levels of ammonia, high levels of valproate, or both [1,9,10]. CSF findings are unremarkable, and currently available evidence is insufficient to associate CSF white cell count and protein levels with VPA-induced encephalopathy [1]. EEG findings in such cases are usually ambiguous, and there are no established EEG diagnostic criteria for valproate-induced encephalopathy [1]. In our patient, generalised cerebral dysfunction without epileptiform activity helped support a toxic-metabolic encephalopathy once structural and infectious aetiologies were excluded.

Valproic acid, often used as an anti-seizure medication, can provoke arrhythmias. In a published case, longer QT intervals were reported in individuals on VPA compared to non-medicated patients [11]. Sinus bradycardia and hypotension are documented in VPA overdose but are rarely seen in therapeutic doses [12]. One study reported reduced atrial ectopic beats after initiation of VPA [13]. A notable and unusual aspect of this case was marked sinus bradycardia occurring concurrently with encephalopathy resolving after valproate cessation.

There’s evidence of benefit of L-carnitine therapy in VPA overdose, but its role in therapeutic serum concentrations remains arguable [9]. In our patient, the key intervention was withholding VPA, which reversed the neurotoxicity, and an absolute return to baseline was achieved.

Limitations to this case report include the confounding effect of simultaneous suspension of VPA and mirtazapine, the absence of measured serum or CSF glutamine levels, no serum L-carnitine assay, and an incomplete autoimmune workup. Furthermore, causality assessment using the Naranjo Adverse Drug Reaction Probability Scale [15] yielded a score of 3, classifying the association between valproate and encephalopathy as a possible adverse drug reaction. This relatively low score reflects the inherent difficulty in establishing a definitive causal relationship in the absence of a rechallenge and given the presence of potential confounding factors.

## Conclusions

This case supports a probable association between valproic acid therapy and the development of encephalopathy and marked sinus bradycardia despite therapeutic serum valproate concentrations and normal ammonia levels. This presentation differs from many previously reported cases of valproate-associated encephalopathy, which are more commonly associated with elevated serum valproate levels and/or hyperammonaemia.

For clinicians involved in acute care, this case highlights the importance of considering valproate-associated toxicity in patients presenting with subacute neurological deterioration and unexplained bradycardia, even when serum valproate concentrations and ammonia levels are within the normal range. Careful review of medication-related adverse effects and potential drug-drug interactions may facilitate earlier recognition, prompt withdrawal of the offending agent(s), and timely management. While the clinical course and improvement following medication withdrawal support a probable association with valproate, a definitive causal relationship cannot be established, particularly as mirtazapine was discontinued concurrently.
